# An Ectopic Pancreatic Mass Inducing Gastric Outlet Obstruction

**DOI:** 10.7759/cureus.87449

**Published:** 2025-07-07

**Authors:** Rohan Ahuja, Samir Mehta, Nakia Armendariz, Jimmy Q Pham, Madhav Desai

**Affiliations:** 1 Internal Medicine, University of Texas Health Science Center at Houston, Houston, USA; 2 Internal Medicine, University of Missouri-Kansas City School of Medicine, Kansas City, USA; 3 Gastroenterology, McGovern Medical School at University of Texas Health Science Center at Houston, Houston, USA; 4 Gastroenterology, Borland Groover Clinic, Jacksonville, USA

**Keywords:** carcinoembryonic antigen (cea), endoscopic ultrasound (eus), gastric outlet obstruction (goo), pancreatic cyst management, ectopic pancreas

## Abstract

Gastric outlet obstruction (GOO) is a clinical diagnosis characterized by early satiety, abdominal pain, post-prandial vomiting, and weight loss. Here, we present a rare case of an ectopic pancreatic cyst inducing GOO, highlighting the importance of a thorough diagnostic workup. A 40-year-old man with no prior medical or surgical history presented with a six-month history of intermittent epigastric pain, nausea, and vomiting with an inability to tolerate oral intake for a week. CT of the abdomen and pelvis revealed a 4.5 × 3.0 × 2.9 cm, well-circumscribed, peripherally enhancing cystic lesion in the distal pylorus. Initial esophagogastroduodenoscopy (EGD) revealed an obstructive submucosal mass at the antrum. Random gastric biopsies were negative for malignancy or evidence of *Helicobacter pylori*. Endoscopic ultrasound (EUS) was performed upon repeat EGD for further characterization of the submucosal lesion, revealing a 5 cm × 5 cm perigastric cyst with anechoic and hypoechoic areas at the site of extrinsic compression in the gastric antrum. Under Doppler guidance, fine-needle aspiration was performed, with dark brown aspirate and pyloric narrowing resolution due to cyst size reduction. Subsequently, the patient’s symptoms significantly improved. Fluid analysis of the aspirate revealed an elevated carcinoembryonic antigen (CEA) level at 736 ng/mL and amylase of 2,113 ng/mL, indicating the nature of the cystic lesion to be ectopic mucinous pancreatic tissue. The mechanism of growth of the cyst was unclear but could be explained by continuous mucinous secretions due to the elevated CEA or evidence of chronic pancreatitis on EUS, leading to continuous extravasation. This case highlights the importance of maintaining a broad differential when evaluating GOO and including ectopic pancreas as a potential etiology.

## Introduction

Gastric outlet obstruction (GOO) is a clinical diagnosis marked by early satiety, abdominal pain, postprandial vomiting, weight loss, and a characteristic succussion splash on examination. Three categories of obstruction include benign, malignant, and gastroparesis-induced GOO [1]. Among benign causes, GOO commonly occurs due to peptic ulcer disease, though it may also result from medication use, toxic ingestion, anatomical obstruction, or inflammatory conditions. Evaluation often includes laboratory assessment of electrolyte abnormalities and acid-base disorders, abdominal X-ray, and esophagogastroduodenoscopy (EGD) with possible endoscopic ultrasound (EUS) [2]. Cysts represent a rare cause of GOO, with perigastric cysts being an even more infrequent cause of obstruction. Ectopic pancreatic tissue, a relatively common incidental finding, is often found in the antrum of the stomach and is generally asymptomatic, though it may be complicated by malignant transformation or anatomic compression due to large size [3]. Here, we present a rare case of an ectopic pancreatic cyst inducing GOO, highlighting the importance of a thorough diagnostic workup. This article was previously presented as a meeting abstract at the 2024 ACG Annual Scientific Meeting on October 27th, 2024.

## Case presentation

**Figure 1:**
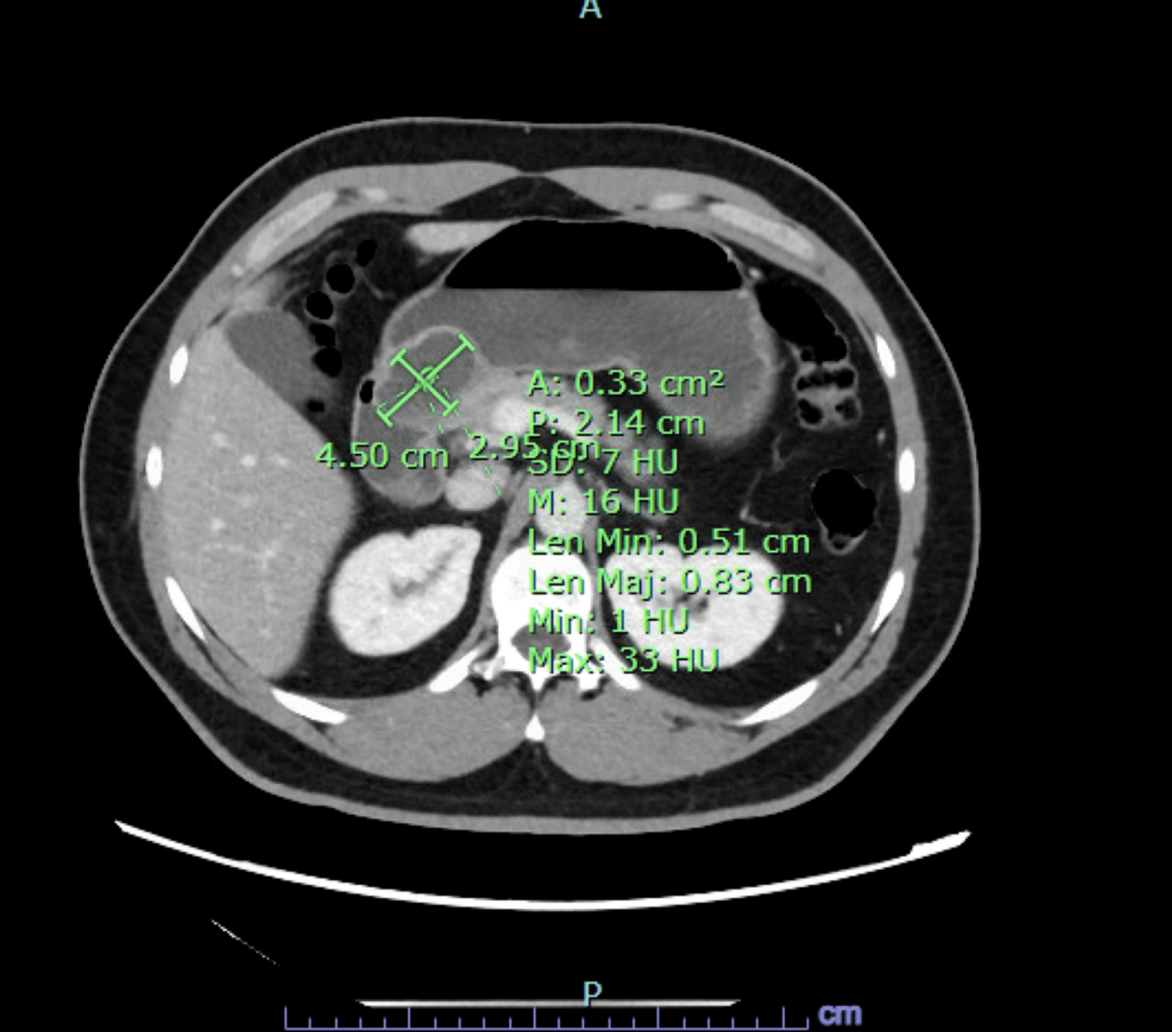
Initial CT identification of gastric outlet obstruction. CT of the abdomen obtained upon initial presentation, revealing a 4.5 × 3.0 × 2.9 cm cystic mass in the distal pylorus.

**Figure 2:**
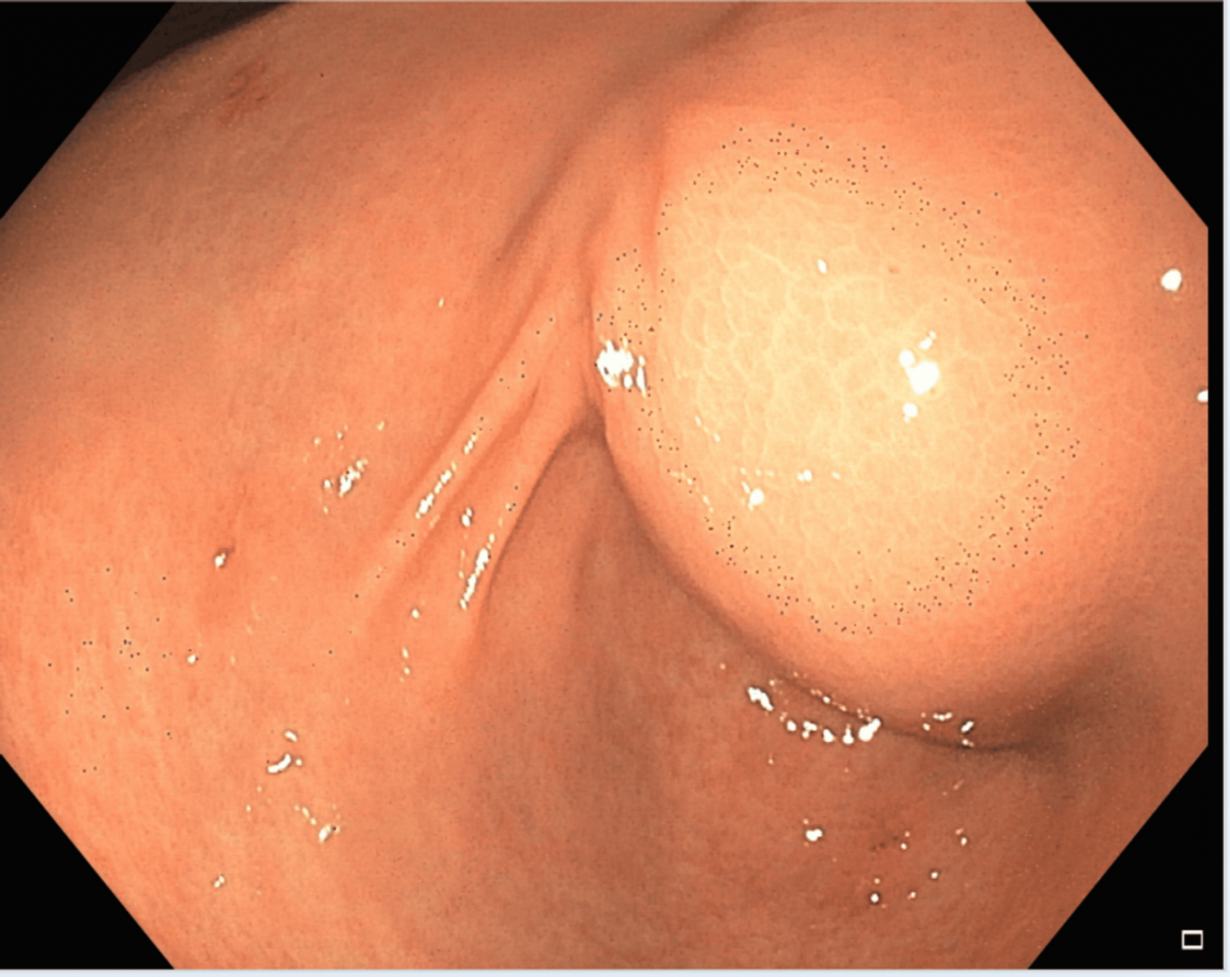
Endoscopic view of the cystic mass. Presence of a large submucosal mass in the antrum, as visualized on endoscopy.

**Figure 3:**
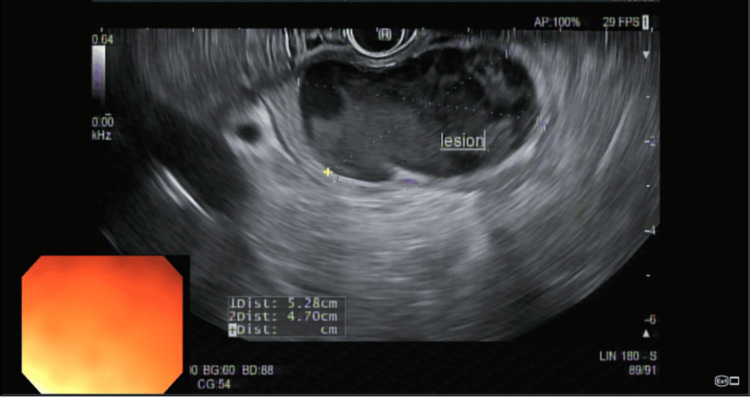
Endoscopic ultrasound view of the cystic mass. Hypoechoic and anechoic areas within a  5 × 5 cm mass seen upon ultrasound of the antrum that was later drained.

A 40-year-old man with no prior medical or surgical history presented with six months of intermittent epigastric pain, nausea, and vomiting with an inability to tolerate oral intake for a week. Laboratory evaluation was only notable for a leukocytosis of 18.2 × 10^3^/µL. All labs on presentation are listed in Table 1. However, CT of the abdomen and pelvis (Figure 1) revealed a 4.5 × 3.0 × 2.9 cm, well-circumscribed, peripherally enhancing cystic lesion in the distal pylorus. Initial EGD (Figure 2) revealed an obstructive submucosal mass at the antrum. Random gastric biopsies were negative for malignancy or evidence of *Helicobacter pylori*. EUS (Figure 3) was performed upon repeat EGD for further characterization of the submucosal lesion, revealing a 5 cm × 5 cm perigastric cyst with anechoic and hypoechoic areas at the site of extrinsic compression in the gastric antrum. Under Doppler guidance, fine-needle aspiration was performed, with dark brown aspirate and resolution of pyloric narrowing due to cyst size reduction. Subsequently, the patient’s symptoms significantly improved. Fluid analysis of the aspirate revealed an elevated CEA level at 736 ng/mL and amylase of 2,113 ng/mL, indicating the nature of the cystic lesion to be ectopic mucinous pancreatic tissue. No further follow-up visits were available for the patient.

**Table 1:** Laboratory values on presentation. Laboratory values from the patient upon presentation to the hospital. Notably, carcinomembryonic antigen was collected, but did not result till later in admission.

Laboratory test	Value (units)	Reference range (units)
Sodium	145 mmol/L	136–145 mmol/L
Potassium	3.8 mmol/L	3.8–5.1 mmol/L
Chloride	102 mmol/L	98–107 mmol/L
Bicarbonate	27 mmol/L	21–31 mmol/L
Blood urea nitrogen	17 mg/dL	7–25 mg/dL
Creatinine	1.0 mg/dL	0.7–1.3 mg/dL
Albumin	5.3 g/dL	4.2–5.5 g/dL
Aspartate aminotransferase	18 U/L	13–39 U/L
Alanine aminotransferase	18 U/L	13–39 U/L
Total bilirubin	0.7 mg/dL	0.2–1.2 mg/dL
Alkaline phosphatase	53 U/L	34–104 U/L
Lipase	29 U/L	11–82 U/L
Carcinoembryonic antigen	783 ng/mL	No reference range
White blood count	18.5 × 10^3^/µL	4.6–12.0 × 10^3^/µL
Red blood count	15.2 g/dL	13.2–16.6 g/dL

## Discussion

Ectopic pancreas most commonly presents in the submucosal layer of the antrum and is typically asymptomatic. It is defined as pancreatic tissue without an anastomotic connection to the original organ [4]. The specific cause of these lesions is unknown; however, the leading theory is that one or more of the duodenal wall evaginations from which the pancreas arises remains in the bowel and migrates to form ectopic pancreatic tissue [5]. Most often, they are asymptomatic collections with an incidence ranging from 0.5% to 13.7% of people, though they have the potential for malignant transformation, acute and chronic inflammatory insults, and pseudocyst formation [6]. Patients can additionally present with epigastric pain, abdominal fullness, dyspepsia, melena, and even gastric/duodenal ulceration [7]. This is a rare case of an ectopic pancreatic mass leading to GOO. The mechanism of growth of the cyst is unclear, but it can be explained by continuous secretions of mucin, given the elevated CEA. Two reports described similar cases of GOO caused by submucosal cystic masses originating as sequelae of chronic pancreatitis of the ectopic pancreatic masses [8,9]. Management of this condition can vary based on the size of the lesion, symptom burden, and the clarity of the diagnosis. In our patient, resolution was achieved with simple drainage of the cystic fluid; however, surgical resection may be warranted in cases where malignancy is suspected or in repeated cyst recurrence. Differentiation between cyst and stromal tumors, leiomyomas, adenomatous polyps, and metastatic disease is challenging without EUS and tissue sampling. Few case reports exist of ectopic pancreatic masses leading to GOO in the available literature, with EUS performed most often for diagnostic and therapeutic evaluation. Given the lack of ability to differentiate between a benign and malignant condition, EUS with tissue acquisition should be performed for all submucosal masses. In one case series evaluating ectopic pancreas in the stomach, 13 out of the 21 patients were determined to have adenocarcinoma upon histologic sampling [10].

## Conclusions

While large trials and data are not available, it is crucial to consider GOO when evaluating patients with classic symptoms and suggestive EGD findings. Although an abnormal etiology for GOO, an ectopic pancreatic cyst should be considered in the differential diagnosis. Ectopic pancreatic tissue is uncommon in the general population, but the development of a cyst large enough to obstruct the gastric outlet is an even more unique scenario not well documented in the literature. EUS represents the best clinical modality for diagnostic and therapeutic evaluation of pancreatic cysts, which was appropriately used in this clinical scenario. More information is needed regarding the optimal follow-up and risk of malignant transformation. As further cases appear in the literature, more information can be derived regarding risk factors and prevention for this cohort of patients.
